# Supplementary material for: RNAi epimutations conferring antifungal drug resistance are inheritable
Source: Nat Commun. 2025 Aug 7;16:7293. doi: 10.1038/s41467-025-62572-6 (PMC12332000; doi:10.1038/s41467-025-62572-6)
Supplement: Supplementary file 2 — Description of Additional Supplementary Files [file 41467_2025_62572_MOESM2_ESM.pdf]

Description of Additional Supplementary Files:

Supplementary Data 1: Small RNAproducing loci identified in PS15– (PS15m) and PS15+ (PS15p). Dataset shows locus coordinates, name, length, as well as the absolute number of Reads, quantification in RPM, fraction of reads from the major RNA species (FracTop), strand orientation, major RNA sequence and its read count, sequence complexity, Dicer processing prediction, miRNA annotation, phasing score, and counts of reads by size class (21–25 nt).

Supplementary Data 2: Variant dataset including scaffold (CHROM), position (POS), variant ID, reference (REF) and alternate (ALT) alleles, manual curation notes (Fail or Pass), Isolates with the variant, quality score (QUAL), GATK4 filter status (Fail or Pass), variant annotations (INFO), genotype format, genotypes for samples, gene name affected, predicted consequence, impact level, and detailed variant positions within cDNA, CDS, and protein, including amino acid and codon changes, distance to nearest feature, and strand orientation.

Supplementary Data 3:

o Diffbind analysis. RNA Polymerase II differential binding regions with scaffold (seqnames), start and end coordinates, region width, strand, normalized binding intensities in combined (Conc), epimutant (Conc_Epi), and naive wild type (Conc_Naive) samples, fold change (Fold), statistical significance (p.value and FDR), and peak presence calls in each condition (Called1, Called2).

o Peak calling datasets. RNA Polymerase II called peaks with scaffold (chr), start and end coordinates, peak length, absolute summit position within the peak (abs_summit), pileup height, significance scores (– log10 p-value and –log10 qvalue), fold enrichment over background, and peak name.

Supplementary Data 4: Differential expression results including gene ID, mean normalized expression (baseMean), log2 fold change (Epimutant vs WT), standard error of fold change (lfcSE), test statistic (stat), p-value (pvalue), adjusted pvalue (padj), and log2 normalized counts for the listed isolates.

Supplementary Data 5: Differential expression results including gene ID, mean normalized expression (baseMean), log2 fold change (Epimutant vs WT), standard error of fold change (lfcSE), test statistic (stat), p-value (pvalue), adjusted pvalue (padj), and log2 normalized counts for the listed isolates.

Supplementary Data 6: Sequencing metadata including SRA accession (SRR Acc.), BioProject and BioSample accessions, library identifier, read layout (e.g., paired or single-end), sequencing platform, sequencing type, isolate name, and publication.
