# Supplementary material for: RNAi epimutations conferring antifungal drug resistance are inheritable
Source: Nat Commun. 2025 Aug 7;16:7293. doi: 10.1038/s41467-025-62572-6 (PMC12332000; doi:10.1038/s41467-025-62572-6)
Supplement: Supplementary file 9 — Reporting Summary [file 41467_2025_62572_MOESM9_ESM.pdf]

nature portfolio

Corresponding author(s): Joseph Heitman, Carlos Perez-Arques, Maria Isabel Navarro-Mendoza

Last updated by author(s): Jul 11, 2025

Reporting Summary

Nature Portfolio wishes to improve the reproducibility of the work that we publish. This form provides structure for consistency and transparency in reporting. For further information on Nature Portfolio policies, see our Editorial Policies and the Editorial Policy Checklist.

Statistics

For all statistical analyses, confirm that the following items are present in the figure legend, table legend, main text, or Methods section.

n/a	Confirmed	
☐	☒	The exact sample size (*n*) for each experimental group/condition, given as a discrete number and unit of measurement
☐	☒	A statement on whether measurements were taken from distinct samples or whether the same sample was measured repeatedly
☐	☒	The statistical test(s) used AND whether they are one- or two-sided<br>*Only common tests should be described solely by name; describe more complex techniques in the Methods section.*
☒	☐	A description of all covariates tested
☐	☒	A description of any assumptions or corrections, such as tests of normality and adjustment for multiple comparisons
☐	☒	A full description of the statistical parameters including central tendency (e.g. means) or other basic estimates (e.g. regression coefficient) AND variation (e.g. standard deviation) or associated estimates of uncertainty (e.g. confidence intervals)
☐	☒	For null hypothesis testing, the test statistic (e.g. *F*, *t*, *r*) with confidence intervals, effect sizes, degrees of freedom and *P* value noted<br>*Give P values as exact values whenever suitable.*
☒	☐	For Bayesian analysis, information on the choice of priors and Markov chain Monte Carlo settings
☒	☐	For hierarchical and complex designs, identification of the appropriate level for tests and full reporting of outcomes
☐	☒	Estimates of effect sizes (e.g. Cohen's *d*, Pearson's *r*), indicating how they were calculated

Our web collection on statistics for biologists contains articles on many of the points above.

Software and code

Policy information about availability of computer code

Data collection	No software was utilized for data collection
Data analysis	Genome assembly, annotation, and homolog identification<br>Basecalling was performed on MinION raw signal-level, POD5 output using ONT Dorado v0.7.0 (https://github.com/ nanoporetech/dorado/) basecaller, selecting the super-accuracy sup@v3.6 basecalling model and the EXP-NBD104 kit (--kit-name). Basecalled reads were demultiplexed and trimmed into PS15– and + high quality FASTQ reads with Dorado modules demux and trim. Draft genome assemblies were generated using Flye v2.9.3-b1797 50 for high-quality reads (--nano-hq), setting 0.05 estimated per-read sequencing error rate (--read-error 0.05) and excluding alternative contigs (--no-alt-contigs). To optimize assembly quality, several runs were performed with varying minimum overlap lengths (--min-overlap) ranging from 5,000 to 10,000 base pairs in 1,000 bp intervals. For each mating type, the assembly exhibiting the highest completeness and contiguity was selected for downstream processing. Genome assemblies were iteratively polished using Racon v1.5.0 51 with default settings until no substantial improvements were observed between successive iterations. Further error correction was performed with default NextPolish1 v1.4.1 52 and 2 v0.2.0 53 using long, ONT-based and Illumina-based, adapter-trimmed short reads. Long reads were aligned with Minimap2 v2.26-r1175 54 default parameters for ONT reads (-ax map-ont), as well as short reads with BWA-MEM v0.7.17-r1188 55 default settings, to examine coverage as a measure of assembly correctness. NCBI contamination detection pipeline was run on the assemblies to identify and remove potential contaminant sequences, including vector, adapter, and non-target organism sequences, following NCBI's standard submission guidelines.<br>Contig sequences from both mating type assemblies were aligned with Minimap2 (-x asm5 -N 0 --secondary no) and visualized using Circos v0.69-8 56 to examine synteny between both assemblies (Extended Data Fig. 4). 13 alignment blocks that showed perfect synteny and contiguity between both assemblies –referred to as synteny blocks– were selected for meiotic recombination analyses.<br>Repeated sequences were identified utilizing RepeatModeler2 v2.0.5 57 and default parameters, and classified with RepeatMasker v4.1.6 (http://www.repeatmasker.org/) by performing iterative similarity searches against the RepBase database for RepeatMasker

(Edition-20181026) 58 and already classified repeats into repeat libraries for each mating type; after that, genome assemblies were masked by RepeatMasker using these repeat libraries. Gene annotation was conducted using the Funannotate pipeline v1.8.15 59 with strain-specific training models generated from our RNA data, including the clean, train, predict, and update modules. Functional annotation of the proteomes was predicted using eggNOG emapper v2.1.12 (--go_evidence all) and InterProScan 5.73-104.0 60 (options --goterms, --pathways, --iprlookup enabled). The resulting annotations were integrated into the genome assembly using the Funannotate annotate module.

Genome assemblies were evaluated using QUAST v5.2.0 61 for contiguity metrics and the eukaryotic ortholog database from BUSCO v5.7.1 62 for completeness (--lineage eukaryota_odb10, --mode genome, --augustus). Quality metrics are shown in Extended Data Table 1. RNAi components were identified by NCBI BLASTp v1.12.0 63 searches using M. lusitanicus protein sequences as queries, extracted from M. lusitanicus MU402 v1.0 available at the Joint Genome Institute Mycocosm platform (https://mycocosm.jgi.doe.gov/). Identified proteins that also returned positive reciprocal BLASTp hits were retained, and their protein domain configuration was predicted with InterProScan v5.70-102.0 to evaluate their putative function (Extended Data Fig. 1a).

Genetic variation analyses to assess meiotic recombination

Low quality and adapter sequences were removed from genomic DNA raw reads using fastp v0.23.4 64 (--average_qual 20, --length_required 148, --length_limit 0, --trim_poly_g, and --dup_calc_accuracy 1). For subsequent analyses, all samples from either mating type were aligned to the newly generated PS15- genome as the reference unless otherwise stated, to preserve genome coordinate consistency across samples. Processed reads were aligned with BWA-MEM ensuring Picard compatibility (-M). Alignments were further processed using the GATK4 suite v4.4.0.0 65 to sort (gatk SortSam --SORT_ORDER coordinate), mark duplicates (gatk MarkDuplicates) and assign read groups (gatk AddOrReplaceReadGroups --RGPL Illumina and other RG accordingly to ID, library, and sample name). A sequence dictionary for the reference genome was generated using CreateSequenceDictionary. For meiotic recombination analyses, variant calling was performed on PS15– and + wildtype datasets by GATK4 HaplotypeCaller (--sample-ploidy 1). Mixed and symbolic variants were excluded using SelectVariants (--select-type-to-exclude MIXED, --select-type-to-exclude SYMBOLIC). Low quality variants were flagged using VariantFiltration with the following arguments: --filter-expression “QD < 20.0” --filter-name “QD20”, --filter-expression "QUAL < 30.0" --filter-name "QUAL30", --filter-expression "SOR > 3.0" --filter-name "SOR3", --filter-expression "FS > 60.0" --filter-name "FS60", --filter-expression "MQ < 40.0" --filter-name "MQ40", and filtered out along with variants shared between PS15+ and PS15– using SelectVariants (--discordance $PS15+.vcf, --exclude-filtered). Additionally, variants overlapping repetitive sequences were excluded with Bedtools v2.30.0 66 (intersect -v), yielding a high-confidence set of PS15+ specific SNVs from non-repetitive genomic regions. A total of 18,731 SNVs overlapping previously described synteny blocks were identified (bedtools intersect), and used to characterize genetic variation in the progeny datasets. To do so, processed alignments from parent and progeny were piled up against this set of PS15+ specific SNVs using GATK4 GetPileupSummaries, establishing a threshold of ≥ 95% read support towards either reference (PS15–, REF, awk '{ratio = ($3 + 0.001) / ($3 + $4 + $5 + 0.001) if (ratio > 0.95) {print $1, $2 - 1, $2, "REF", ratio, "."}}') or alternate position (PS15+, ALT, awk '{ratio = ($3 + 0.001) / ($3 + $4 + $5 + 0.001) if (ratio < 0.05) {print $1, $2 - 1, $2, "ALT", (1 - ratio), "."}}') to be considered inherited SNVs. Same parent variants closer than 50 kb were merged by bedtools merge (-d 50000) to avoid overplotting during rendering (Extended Data Fig. 5).

To identify potential mutations conferring FK506 and rapamycin resistance, a more stringent variant calling analysis was applied to the processed alignments from parental samples (PS15–, E1– to E6–, PS15+, E10+, E12+, and E15+). Variants were called using GATK4 HaplotypeCaller (--sample-ploidy 1, --max-alternate-alleles 3) and filtered as described above, but with stricter thresholds: --filter-expression "QD < 5.0" --filter-name "QD5", --filter-expression "DP < 60.0" --filter-name "DP60", --filter-expression "QUAL < 300.0" --filter-name "QUAL300", --filter-expression "SOR > 1.0" --filter-name "SOR1", --filter-expression "FS > 0.1" --filter-name "FS01", --filter-expression "MQ < 60.0" --filter-name "MQ60". Variants failing these filters were excluded along those shared with either wildtype control (PS15– or PS15+) using SelectVariants (--discordance $wildtypes.vcf, --exclude-filtered), and those overlapping repetitive sequences (bedtools intersect -v). The resulting high-confidence variants were manually curated by examining the processed alignment from their corresponding wild type; variants already present in the wildtype reads were considered false positives and discarded (Extended Data Fig. 7).

DNA coverage was assessed with Deeptools2 bamCoverage v3.5.4 67 (--binSize 50, --normalizeUsing CPM, --minMappingQuality 1) and coverage plots were centered to approximately whole-genome average coverage, which was assumed to represent a haploid genome (Extended Data Fig. 6).

RNA-sequencing analyses

Raw sRNA reads were processed with UMI-tools (https://github.com/CGATOxford/UMI-tools) to detect the UMI sequences (umi_tools extract --extract-method=regex, --bc-pattern='.+(?P<discard_1>AACTGTAG GCACCATCAAT)(?P<umi_1>.{12})'), and then trimmed with Trim Galore! v0.6.10 (https://github.com/FelixKrueger/ TrimGalore) using stringent parameters (--stringency 4, --quality 20, --max_n -0, -e 0.1, --length 10, --max_length 75) to avoid random 3’-end trimming. Processed reads were aligned using Bowtie v1.3.1 68 (-v 0 -k 1 --best). ShortStack v3.8.5 69 was run on the resulting alignment files to identify sRNA-producing loci agnostically (ShortStack --genomefile $PS15-_genome_assembly.fasta --nohp --dicermin 21 --dicermax 25). Bases of sRNA-producing loci overlapping coding, intergenic, and repetitive regions were quantified using bedtools intersect -wo and visualized as a pie chart (Extended Data Fig. 2a). Additionally, sRNAs mapping to annotated gene features were quantified using ShortStack as previously (adding --locifile $PS15-.gff3).

Alternatively and to assess splice junction read support (Extended Data Fig. 9c), processed reads were aligned with STAR v2.7.11b 70 (--outSAMprimaryFlag AllBestScore, --alignIntronMin 5, --alignIntronMax 200, --outSAMmultNmax 1, --outFilterMultimapNmax 1, --outFilterMismatchNmax 0, --sjdbGTFfile $PS15-.gff3, --sjdbOverhang 22, --sjdbScore 5, --alignSJoverhangMin 2, --alignSJDBoverhangMin 1).

Samtools v1.10 71 and Bioawk v20110810 (https://github.com/lh3/bioawk) were used to count and filter reads harboring specific siRNA features, namely antisense orientation, length of 21-24 nt, and 5’-U. For reads mapping to features on the forward strand, reverse reads were considered antisense (samtools view -L $feature_coordinates.bed -F 4 -f 16 | bioawk -c sam '{if(length($seq) >= 21 && length($seq) <= 24 && substr($seq,length($seq),1) == "A") print }'); for features on the reverse strand, forward reads were retrieved as antisense (samtools view -L $feature_coordinates.bed -F 20 | bioawk -c sam '{if(length($seq) >= 21 && length($seq) <= 24 && substr($seq,1,1) == "T") print }'). Note that in SAM alignments, all sequences are printed in the forward orientation; therefore, aligned 3’-A reverse reads correspond to 5’-U reads. Coverage files were generated using bamCoverage (--binSize 5, --normalizeUsing CPM, --minMappingQuality 0) for both total sRNA (non-filtered alignments) and siRNA (filtered alignments). Relative abundances of siRNA reads were computed ((read number)⁄(total fkbA reads)), and this standardized relative abundances were utilized to perform a PCA to assess similarities among samples (Fig. 3g).

Long RNA raw reads were trimmed with fastp as previously described and aligned with STAR (--outSAMprimaryFlag AllBestScore, --alignIntronMin 5, --alignIntronMax 200, --outSAMmultNmax 100, --outFilterMultimapNmax 100, --sjdbGTFfile $PS15-.gff3, --sjdbOverhang 150, --alignSJoverhangMin 2). For differential expression (DE) analyses, read counts mapping to annotated features were computed using featureCounts (-p, --countReadPairs, -t ‘exon’, -g ‘gene_id’, -F ‘GTF’ -s 2, --fracOverlap 0, fracOverlapFeature 0). Differential expression was then quantified using DESeq2 v1.44.0 72 by applying the estimateSizeFactors, counts, and DESeq functions to the count matrix, followed by a log2 transformation of counts per million (CPM). Expression differences were considered significant at an adjusted p-value or False Discovery Rate below 0.05 (FDR ≤ 0.05). Normalized expression values were visualized in a heatmap generated with ComplexHeatmap v2.20.0 73 (Extended Data Fig. 8d, e).

For genomic visualization, long RNA alignments were split by strand using Samtools. Forward reads were obtained by merging second-in-pair forward reads (samtools view -b -f 128 -F 16) with first-in-pair reverse reads (samtools view -b -f 80). Similarly, reverse reads were obtained by merging second-in-pair reverse reads (samtools view -b -f 144) with first-in-pair forward reads (samtools view -b -f 64 -F 16). Stranded coverage files were generated using bamCoverage (--binSize 5, --normalizeUsing CPM, --minMappingQuality 0).
Chromatin immunoprecipitation-sequencing analyses
Raw read samples were processed with fastp to remove adapter and low-quality sequences (--average_qual 20, --length_required 51, --length_limit 0, --trim_poly_g, --dup_calc_accuracy 1, --detect_adapter_for_pe). Processed reads were aligned with BWA-MEM (-M). ChIP-enriched regions were identified using MACS2 v2.2.9.1 74 (callpeak --extsize 200, --nomodel, --gsize 37441900) with both narrow (--call-summits) and broad (--broad) peak settings. Differential binding analysis was performed using DiffBind 3.14.0 75 , including read quantification and normalization (dba.count, bUseSummarizeOverlaps=TRUE, score=DBA_SCORE_NORMALIZED), contrast definition (dba.contrast, minMembers=2, categories=DBA_FACTOR) and identification of RNA polymerase II binding differences (dba.analyze, method=DBA_ALL_METHODS) between epimutant and wildtype samples, specifically at the fkbA locus (Extended Data Fig. 8a).
Additionally, genes embedded in H3K9me-based heterochromatin were identified using bedtools intersect and H3K9me2 broad peaks (-f 0.9 -u). Shared H3K9me2-embedded genes between epimutant and wildtype samples were visualized as a Venn diagram generated by ggVennDiagram v1.5.2 76 (Extended Data Fig. 8c).
For genomic visualization, coverage of ChIP-enrichment was determined as the IP/Input DNA ratio using Deeptools 2 bamCompare (--binSize 25, --normalizeUsing CPM, --minMappingQuality 0, --operation ratio).

For manuscripts utilizing custom algorithms or software that are central to the research but not yet described in published literature, software must be made available to editors and reviewers. We strongly encourage code deposition in a community repository (e.g. GitHub). See the Nature Portfolio guidelines for submitting code & software for further information.

Data

Policy information about availability of data

All manuscripts must include a data availability statement. This statement should provide the following information, where applicable:

- Accession codes, unique identifiers, or web links for publicly available datasets
- A description of any restrictions on data availability
- For clinical datasets or third party data, please ensure that the statement adheres to our policy

Genome assemblies, gene annotation, and raw ONT and Illumina-based raw FASTQ reads are publicly available under NCBI's Sequence Read Archive (SRA) project accessions PRJNA1168935 (PS15–) and PRJNA1168941 (PS15+). The remaining raw sequencing data can be accessed under PRJNA1170303, including small RNA, rRNA-depleted RNA, ChIP, and Illumina whole-genome sequencing. In addition, M. lusitanicus PS10 wildtype ChIP and small RNA data was retrieved from the publicly available project accession PRJNA903107 and used to compare our findings. Individual SRA run (SSR) accession numbers are listed in Supplementary Data 6. Source data are provided with this paper, including the raw data underlying bar and scatter plots, as well as uncropped and unprocessed scans of Northern blots. These are organized as multiple labeled Tab-separated values (TSV) or PDF files within separate folders in a compressed Source Data ZIP archive.

Research involving human participants, their data, or biological material

Policy information about studies with human participants or human data. See also policy information about sex, gender (identity/presentation), and sexual orientation and race, ethnicity and racism.

Reporting on sex and gender	No human participants or data were involved in this study
Reporting on race, ethnicity, or other socially relevant groupings	No human participants or data were involved in this study
Population characteristics	No human participants or data were involved in this study
Recruitment	No human participants or data were involved in this study
Ethics oversight	No human participants or data were involved in this study

Note that full information on the approval of the study protocol must also be provided in the manuscript.

Field-specific reporting

Please select the one below that is the best fit for your research. If you are not sure, read the appropriate sections before making your selection.

[x] Life sciences [ ] Behavioural & social sciences [ ] Ecological, evolutionary & environmental sciences

For a reference copy of the document with all sections, see nature.com/documents/nr-reporting-summary-flat.pdf

Life sciences study design

All studies must disclose on these points even when the disclosure is negative.

Sample size	For progeny analysis, no sample-size calculation was performed. Sample size was determined according to empirical limitations and feasibility, i.e. ~1% zygospore germination rate. Initially, we aimed at 500 zygospores per cross, resulting in 3-10 germinating zygospores.<br>For ChIP qPCR, technical triplicates were selected as sample size according to standards in the field. The number of replicates used per experiment is reported in the figure legends and Methods section, and are justified based on reproducibility and statistical robustness.

Data exclusions	No data were excluded from the analyses
Replication	Small RNA and ChIP sequencing were performed in several biological replicates, including different species and opposite mating types
Randomization	Group randomization was not needed as we analyzed every progeny resulting from genetic crosses. When needed, we selected representative samples for further sequencing (small RNA or ChIP) at random.
Blinding	Blinding was not possible and is not relevant to the study, as determining mating type and drug resistance was needed for every parent and progeny used in the study.

Reporting for specific materials, systems and methods

We require information from authors about some types of materials, experimental systems and methods used in many studies. Here, indicate whether each material, system or method listed is relevant to your study. If you are not sure if a list item applies to your research, read the appropriate section before selecting a response.

Materials & experimental systems

n/a	Involved in the study	
☐	☒	Antibodies
☒	☐	Eukaryotic cell lines
☒	☐	Palaeontology and archaeology
☒	☐	Animals and other organisms
☒	☐	Clinical data
☒	☐	Dual use research of concern
☒	☐	Plants

Methods

n/a	Involved in the study	
☐	☒	ChIP-seq
☒	☐	Flow cytometry
☒	☐	MRI-based neuroimaging

Antibodies

Antibodies used	Undiluted ChIP-grade monoclonal antibodies α-H3K9me2 [supplied by Abcam, catalog ab1220, clone mAbcam 1120, lot 1066793-4 of Anti-Histone H3 (di methyl K9) antibody (mAbcam 1220) - ChIP Grade] and α-RNA pol II [supplied by Active Motif, catalog 39497, clone 4H8, lot 23331141 of RNA pol II antibody (mAb)] were utilized to immunoprecipitate chromatin-bound DNA.
Validation	Antibodies are validated by the manufacturer, and also previous publications (https://doi.org/10.1073/pnas.2220475120)

Plants

Seed stocks	No plant species were used for this study
Novel plant genotypes	No plant species were used for this study
Authentication	No plant species were used for this study

ChIP-seq

Data deposition

☒ Confirm that both raw and final processed data have been deposited in a public database such as GEO.

☒ Confirm that you have deposited or provided access to graph files (e.g. BED files) for the called peaks.

Data access links<br>*May remain private before publication.*	Raw reads, including small RNA, mRNA, ChIP, and WGS data are available through the following reviewer link https://dataview.ncbi.nlm.nih.gov/object/PRJNA1170303?reviewer= ir6sn54rqvqdc3cua35r9q71ee<br>In addition, peak calling spreadsheets are included in Supplementary Data 3 and 4, and also through the following NCBI GEO accession link: https://www.ncbi.nlm.nih.gov/geo/query/acc.cgi?acc=GSE280014 using the reviewer token: qvcdekoaxbyxfez
Files in database submission	All SRR files and brief descriptors are listed in Supplementary Data 6<br><br>| SRR Acc. | BioProject Acc. | BioSample Acc | Library ID | Read layout | Platform | Isolate | |-------------|-----------------|----------------|------------------------|-------------|----------|---------|

SRR30945757	PRJNA1170303	SAMN44245086	PS15p_ChIP_K9me2	Paired	Illumina	PS15+
SRR30945756	PRJNA1170303	SAMN44245086	PS15p_ChIP_RNAP	Paired	Illumina	PS15+
SRR30945758	PRJNA1170303	SAMN44245086	PS15p_Input	Paired	Illumina	PS15+
SRR33648455	PRJNA1170303	SAMN48630345	PS15m_ChIP_K9me2	Paired	Illumina	PS15-
SRR33648454	PRJNA1170303	SAMN48630345	PS15m_ChIP_RNAP	Paired	Illumina	PS15-
SRR33648453	PRJNA1170303	SAMN48630345	PS15m_Input	Paired	Illumina	PS15-
SRR30951490	PRJNA1170303	SAMN44253517	PS14WT_ChIP_K9me2	Paired	Illumina	PS14WT
SRR30951489	PRJNA1170303	SAMN44253517	PS14WT_ChIP_RNAP	Paired	Illumina	PS14WT
SRR30951488	PRJNA1170303	SAMN44253517	PS14WT_Input_K9me2	Paired	Illumina	PS14WT
SRR30951487	PRJNA1170303	SAMN44253517	PS14WT_Input_RNAP	Paired	Illumina	PS14WT
SRR30951777	PRJNA1170303	SAMN44253798	PS14E3_ChIP_K9me2	Paired	Illumina	PS14E3
SRR30951776	PRJNA1170303	SAMN44253798	PS14E3_ChIP_RNAP	Paired	Illumina	PS14E3
SRR30951775	PRJNA1170303	SAMN44253798	PS14E3_Input_K9me2	Paired	Illumina	PS14E3
SRR30951774	PRJNA1170303	SAMN44253798	PS14E3_Input_RNAP	Paired	Illumina	PS14E3
SRR22390079	PRJNA903107	SAMN31784349	PS10WT_H3K9me2_Input	Paired	Illumina	PS10WT
SRR22390063	PRJNA903107	SAMN31784349	PS10WT_H3K9me2_IP	Paired	Illumina	PS10WT
SRR22390059	PRJNA903107	SAMN31784349	PS10WT_H3K9me3_IP	Paired	Illumina	PS10WT
SRR22390093	PRJNA903107	SAMN31784349	PS10WT_H3K9me3RNAP_Input	Paired	Illumina	PS10WT
SRR22390110	PRJNA903107	SAMN31784349	PS10WT_RNAP_IP	Paired	Illumina	PS10WT
SRR30951781	PRJNA1170303	SAMN44253835	PS10E1_ChIP_K9me2	Paired	Illumina	PS10E1
SRR30951780	PRJNA1170303	SAMN44253835	PS10E1_ChIP_RNAP	Paired	Illumina	PS10E1
SRR30951779	PRJNA1170303	SAMN44253835	PS10E1_Input_K9me2	Paired	Illumina	PS10E1
SRR30951778	PRJNA1170303	SAMN44253835	PS10E1_Input_RNAP	Paired	Illumina	PS10E1
SRR30943889	PRJNA1170303	SAMN44242504	E10p_ChIP_K9me2	Paired	Illumina	E10+
SRR30943888	PRJNA1170303	SAMN44242504	E10p_ChIP_RNAP	Paired	Illumina	E10+
SRR30943890	PRJNA1170303	SAMN44242504	E10p_Input	Paired	Illumina	E10+
SRR33648458	PRJNA1170303	SAMN48630344	E1m_ChIP_K9me2	Paired	Illumina	E1-
SRR33648457	PRJNA1170303	SAMN48630344	E1m_ChIP_RNAP	Paired	Illumina	E1-
SRR33648456	PRJNA1170303	SAMN48630344	E1m_Input	Paired	Illumina	E1-
SRR30943744	PRJNA1170303	SAMN44242373	#34_ChIP_K9me2	Paired	Illumina	#34
SRR30943743	PRJNA1170303	SAMN44242373	#34_ChIP_RNAP	Paired	Illumina	#34
SRR30943745	PRJNA1170303	SAMN44242373	#34_Input	Paired	Illumina	#34
SRR33648485	PRJNA1170303	SAMN48630343	#10_ChIP_K9me2	Paired	Illumina	#10
SRR33648481	PRJNA1170303	SAMN48630343	#10_ChIP_RNAP	Paired	Illumina	#10
SRR33648482	PRJNA1170303	SAMN48630343	#10_Input	Paired	Illumina	#10

Genome browser session
(e.g. UCSC)

Not applicable. Newly assembled fungal genome not available at UCSC genome browser.

Methodology

Replicates

ChIP-seq was performed on distinct biological samples, which served as biological replicates of either wild types or epimutants. In addition, two biological replicates were prepared per sample, and their DNAs pooled together before library preparation.

Sequencing depth

Sample	Total	Uniquely mapped
PS15m_Input	20623748	17342453
PS15m_ChIP_K9me2	29733618	18972814
PS15m_ChIP_RNAP	3078720	2432710
PS15p_Input	70009712	63783018
PS15p_ChIP_K9me2	30644242	20543856
PS15p_ChIP_RNAP	3631244	1727901
R10p93_Input	65284568	55509549
R10p93_ChIP_K9me2	34120702	27417185
R10p93_ChIP_RNAP	41113750	21352469
R10p_Input	76752346	70654391
R10p_ChIP_K9me2	61008578	58916455
R10p_ChIP_RNAP	19288640	15967063
R1m58_Input	66090636	62666644
R1m58_ChIP_K9me2	27690216	20727464
R1m58_ChIP_RNAP	2650066	2193808
R1m_Input	35590926	28002890
R1m_ChIP_K9me2	37928076	23338152
R1m_ChIP_RNAP	37589040	15215884
PS10WT_ChIP_K9me2	17937064	8830887
PS10WT_ChIP_RNAP	9676396	5722714
PS10WT_Input_K9me2	6046008	2937055
PS10WT_Input_RNAP	8190816	4125584
PS10E1_ChIP_K9me2	13091690	5354170

PS10E1_ChIP_RNAP	9095094	3959828
PS10E1_Input_K9me2	5690050	2841981
PS10E1_Input_RNAP	4702766	2331271
PS14WT_ChIP_K9me2	11749170	4448785
PS14WT_ChIP_RNAP	7507602	2784254
PS14WT_Input_K9me2	8922652	5729109
PS14WT_Input_RNAP	3768376	1657851
PS14E3_ChIP_K9me2	20904566	8570920
PS14E3_ChIP_RNAP	5312668	2291551
PS14E3_Input_K9me2	5735252	2506161
PS14E3_Input_RNAP	6257430	2733886

Antibodies

Undiluted ChIP-grade monoclonal antibodies α-H3K9me2 [supplied by Abcam, catalog ab1220, clone mAbcam 1120, lot 1066793-4 of Anti-Histone H3 (di methyl K9) antibody (mAbcam 1220) - ChIP Grade] and α-RNA pol II [supplied by Active Motif, catalog 39497, clone 4H8, lot 23331141 of RNA pol II antibody (mAb)]

Peak calling parameters

For alignment: bwa mem -t 36 -M $assembly $R1_reads $R2_reads

For peak calling: macs2 callpeak -t $antibody_bamfile -c $input_bamfile -g $effective_genome_size -n $prefix_name --keep-dup all --extsize 200 --nomodel --broad --outdir ./$output_dir

Data quality

Raw data was preprocessed to remove low quality (Q < 20) and short (size < 90 bp) reads. Adapters were also trimmed. Because our aim was to rule out H3K9me2 at epimutational loci, we enforced non-restrictive threshold of FDR >= 0.05 and fold-enrichment >= 2 to call peaks

Software

fastp was used to trim low quality and/or short reads and adapters sequences. BWA-MEM was used to align the data. Deeptools2 bamCompare was used to generate fold-enrichment coverage tracks. MACS2 was used to call broad peaks. Figures were rendered with pyGenomeTracks. For specific versions, arguments, and other details, please review the Methods section.
