# Supplementary material for: RNAi epimutations conferring antifungal drug resistance are inheritable
Source: Nat Commun. 2025 Aug 7;16:7293. doi: 10.1038/s41467-025-62572-6 (PMC12332000; doi:10.1038/s41467-025-62572-6)
Supplement: Supplementary file 11 — Source data [file 41467_2025_62572_MOESM11_ESM.zip › SupplementaryFig6h/sourceDataSupplementaryFig1gFig6hFkbANorthernBlot.pdf]

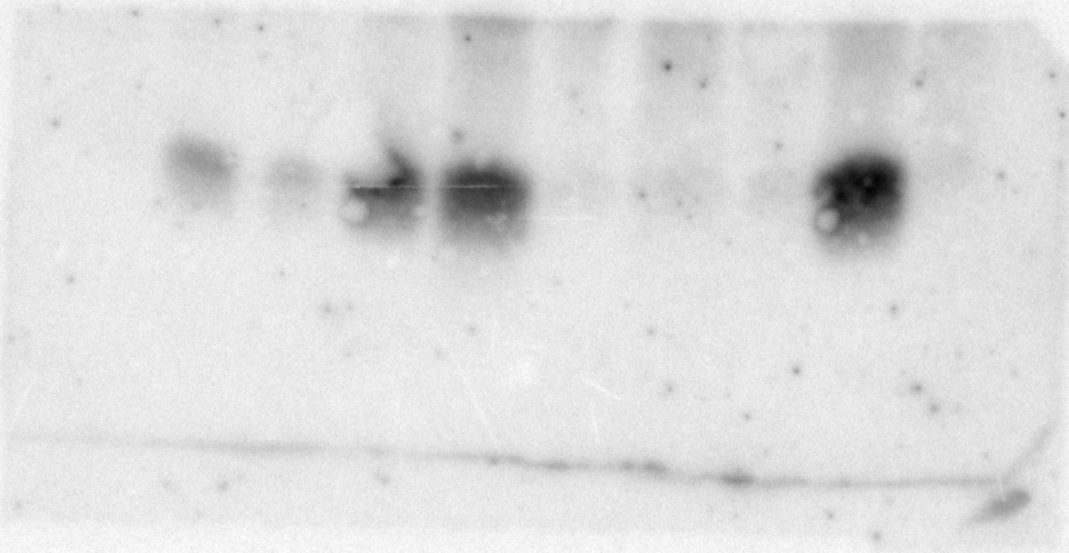

| M1- -control | E15+ Epi | E12+ Epi | E10+ Epi | PS10 E1 +control | PS15+ WT | #13 Epi | #13 Rev | #14 Epi | #14 Rev |
|---|---|---|---|---|---|---|---|---|---|

**fkbA antisense small RNAs**
**blot #2**

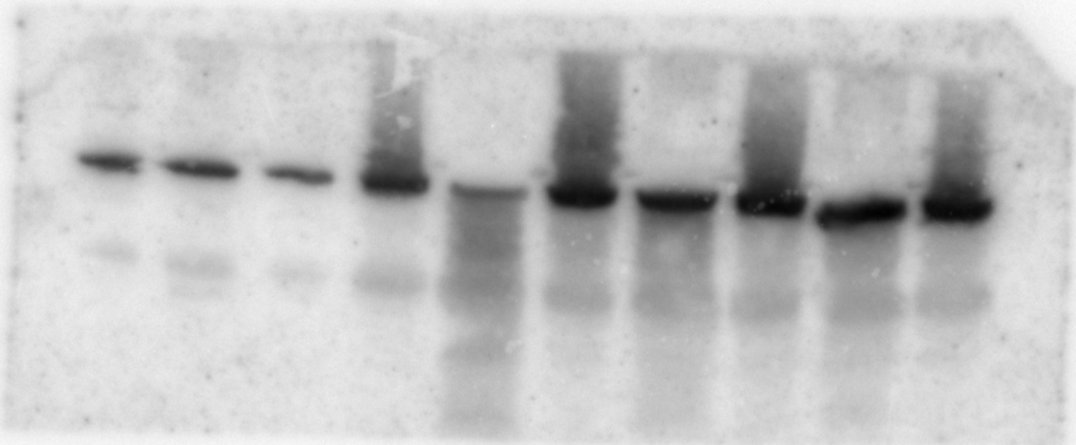

| M1- -control | E15+ Epi | E12+ Epi | E10+ Epi | PS10 E1 +control | PS15+ WT | #13 Epi | #13 Rev | #14 Epi | #14 Rev |
|---|---|---|---|---|---|---|---|---|---|

**rRNA loading control**
**blot #2**
