# Supplementary figures and images for: RNAi epimutations conferring antifungal drug resistance are inheritable
Source: Nat Commun. 2025 Aug 7;16:7293. doi: 10.1038/s41467-025-62572-6 (PMC12332000; doi:10.1038/s41467-025-62572-6)

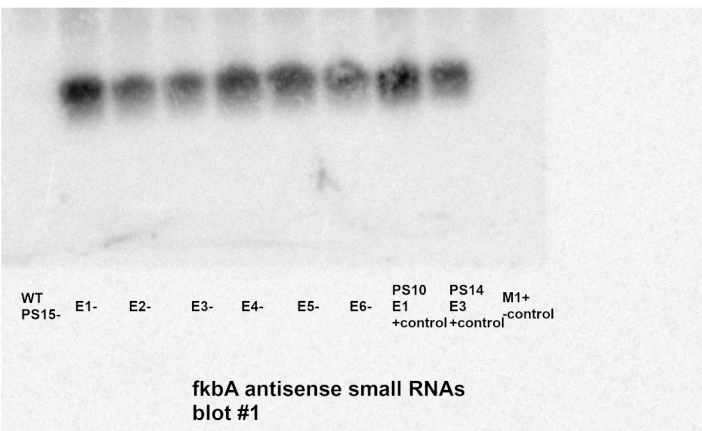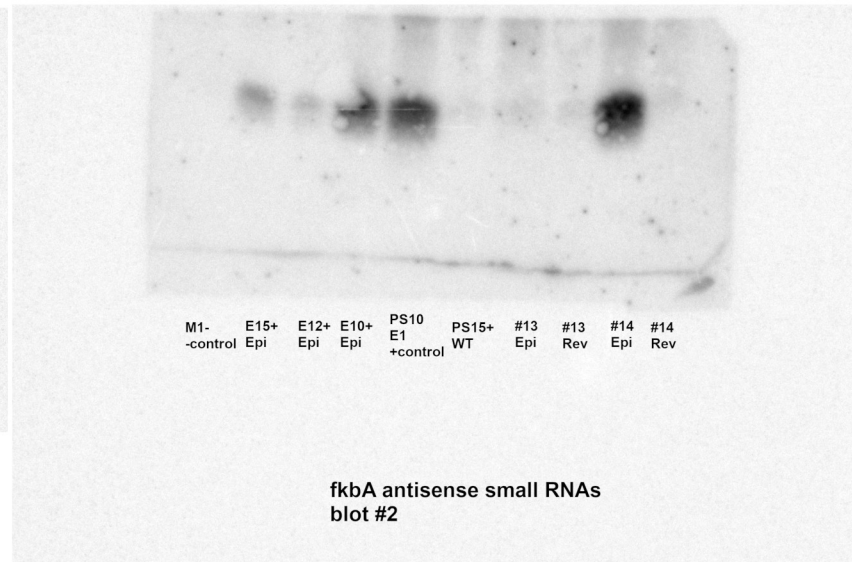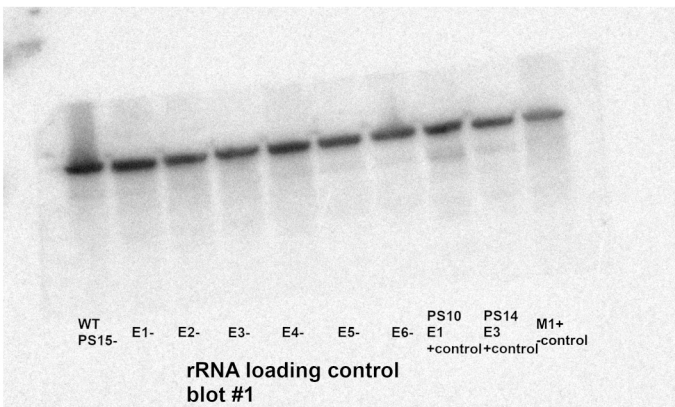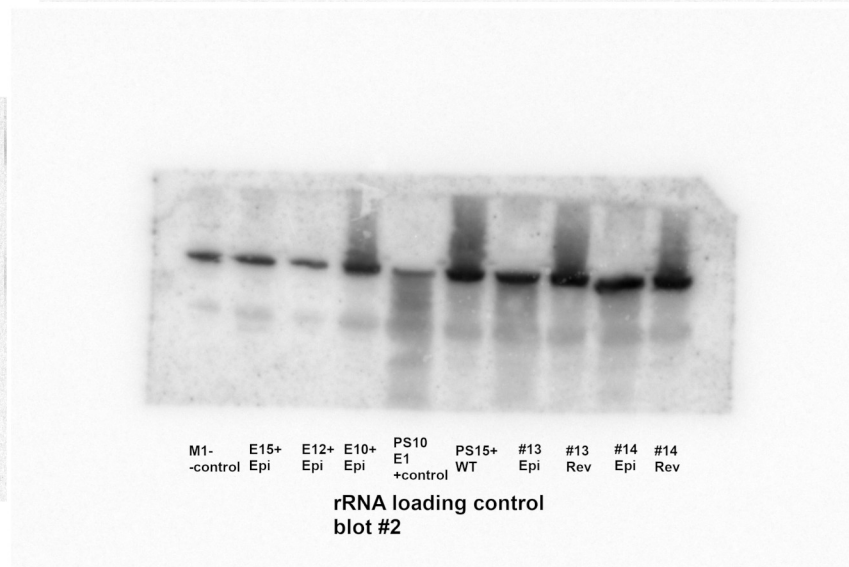

Supplement: Supplementary file 11 — Source data [file 41467_2025_62572_MOESM11_ESM.zip › SupplementaryFig1g/sourceDataSupplementaryFig1g.pdf]
